# Skin microbiome of beluga whales: spatial, temporal, and health-related dynamics

**DOI:** 10.1186/s42523-020-00057-1

**Published:** 2020-10-22

**Authors:** Amy M. Van Cise, Paul R. Wade, Caroline E. C. Goertz, Kathy Burek-Huntington, Kim M. Parsons, Tonya Clauss, Roderick C. Hobbs, Amy Apprill

**Affiliations:** 1 Woods Hole Oceanographic Institution, Woods Hole, MA USA; 2 North Gulf Oceanic Society, Visiting Scientist at Northwest Fisheries Science Center, NOAA National Marine Fisheries Service, Seattle, WA USA; 3 Marine Mammal Laboratory, Alaska Fisheries Science Center, NOAA National Marine Fisheries Service, Seattle, WA USA; 4 Alaska SeaLife Center, Seward, AK USA; 5 Alaska Veterinary Pathology Service, Eagle River, AK USA; 6 Conservation Biology Division, Northwest Fisheries Science Center, NOAA National Marine Fisheries Service, Seattle, WA USA; 7 Animal & Environmental Heath, Georgia Aquarium, Atlanta, GA USA; 8 Marine Mammal Laboratory (retired), Alaska Fisheries Science Center, NOAA National Marine Fisheries Service, Seattle, WA USA

**Keywords:** Conservation, Health assessment, Beluga, Microbiome, 16S rRNA gene

## Abstract

**Background:**

Host-specific microbiomes play an important role in individual health and ecology; in marine mammals, epidermal microbiomes may be a protective barrier between the host and its aqueous environment. Understanding these epidermal-associated microbial communities, and their ecological- or health-driven variability, is the first step toward developing health indices for rapid assessment of individual or population health. In Cook Inlet, Alaska, an endangered population of beluga whales (*Delphinapterus leucas*) numbers fewer than 300 animals and continues to decline, despite more than a decade of conservation effort. Characterizing the epidermal microbiome of this species could provide insight into the ecology and health of this endangered population and allow the development of minimally invasive health indicators based on tissue samples.

**Results:**

We sequenced the hypervariable IV region of bacterial and archaeal SSU rRNA genes from epidermal tissue samples collected from endangered Cook Inlet beluga whales (*n* = 33) and the nearest neighboring population in Bristol Bay (*n* = 39) between 2012 and 2018. We examined the sequences using amplicon sequence variant (ASV)-based analyses, and no ASVs were associated with all individuals, indicating a greater degree of epidermal microbiome variability among beluga whales than in previously studied cetacean species and suggesting the absence of a species-specific core microbiome. Epidermal microbiome composition differed significantly between populations and across sampling years. Comparing the microbiomes of Bristol Bay individuals of known health status revealed 11 ASVs associated with potential pathogens that differed in abundance between healthy individuals and those with skin lesions or dermatitis. Molting and non-molting individuals also differed significantly in microbial diversity and the abundance of potential pathogen-associated ASVs, indicating the importance of molting in maintaining skin health.

**Conclusions:**

We provide novel insights into the dynamics of Alaskan beluga whale epidermal microbial communities. A core epidermal microbiome was not identified across all animals. We characterize microbial dynamics related to population, sampling year and health state including level of skin molting. The results of this study provide a basis for future work to understand the role of the skin microbiome in beluga whale health and to develop health indices for management of the endangered Cook Inlet beluga whales, and cetaceans more broadly.

## Introduction

Although marine mammals are in constant contact with a diverse and abundant seawater microbial community [1], they host their own unique [2], temporally stable [3], and species-specific [4, 5] epidermal microbial communities. These microbes may play an important role in or be indicators of marine mammal health and ecology [6, 7]. As an emerging field of study, marine mammal microbiomes considers these previously overlooked interactions between hosts and their microbiomes [8], and explores how spatial and temporal variability in microbial communities may help to explain the way marine mammal hosts interact with their environment [9].

For many mammals, their associated microbial communities frequently correlate with host health [10]; epidermal microbiomes are similarly thought to be correlated with individual health and disease. In some cases, the presence or absence of skin diseases may depend on the ability of the host microbial community to fight off pathogens [11]. Skin microbiome research has largely focused on humans and humanized model systems [12–14], and much less is known about the skin microbiomes of wildlife. Certain microorganisms may be useful indicators of health, which could help wildlife managers better understand the health of endangered or recovering individuals or populations. However, answering high-resolution questions about microbiomes and health first requires a baseline understanding of the core microbial community associated with a species, and environmentally driven spatio-temporal variability in community composition.

Beluga whales (*Delphinapterus leucas*) are a highly social and gregarious species, forming groups of two to 25 individuals, and, in some populations, aggregating in larger groups (hundreds to thousands) during the summer months [15, 16]. Their complex social structure includes dynamic group affiliations, both with close kin and non-kin, forming large-scale communities comprising both sexes and all age groups [17]. Populations occupying areas of high winter ice cover undergo long-range seasonal migrations to ice-free areas; populations outside the high Arctic do not necessarily undergo similar long range movements, but their movements are often closely linked to available prey, such as salmonids returning to breed in certain river systems [18]. Although fish makes up a large portion of their diet, they are also known to eat benthic invertebrates from shallow estuaries, as well as deep-sea squid and other invertebrates [19]. Longer migrations by arctic beluga whale populations may be related to their apparent need for warmer water temperatures for their annual summer molt [20, 21], similar to other polar populations of marine mammals [22–24], which are thought to be important to maintaining skin health. The annual molt occurs in contrast to most non-polar cetaceans, in which epidermal turnover is a continuous process, and is thought to be partly due to colder water temperatures that slow the breakdown of intercellular lipids and allow superficial cells to remain attached for longer [20]. In beluga whales, microorganisms have been visually observed to be abundant in dead or damaged tissue that remains attached to the surface before the annual molt [20]. The epidermal microbiome of a beluga whale is likely to reflect its complex life history, annual molting cycle, and highly social behavior, as well as the rapidly changing arctic and sub-arctic environments in which it lives.

Bristol Bay and Cook Inlet are both shallow estuarine bays with strong tidal influences, each comprising multiple large river systems that are spawning grounds for several large runs of salmonids, and are home to the two most southerly beluga populations in Alaska. Both populations remain resident to these tidal estuaries throughout the year and feed on flooded tidal plains in the summer, moving out of the riverine and shallow nearshore areas and into deeper water when ice begins to form [25–29]. In both populations, Pacific salmon (*Oncorhynchus* spp.) make up the main portion of the diet during May through October, supplemented in differing proportions by smelt, cod, and invertebrates (primarily shrimp) [19]. Bristol Bay is remote and has remained relatively free of anthropogenic impact over the years, aside from a flourishing salmon fishery [30], while Cook Inlet has seen an increase in human-caused disturbances due to rapid industrial growth along the coast, which is home to approximately half of Alaska’s population, an international airport, the largest vessel port in the state, and a military base, and is downstream of Alaska’s largest agricultural region [31–33]. These activities have led to increased ocean noise and degraded water quality within Cook Inlet, as well as potential *impacts* on prey availability, among other threats [27, 30, 33, 34].

While the size of the beluga population in Bristol Bay is approximately 2000 individuals and has been steady or increasing [35–37], the population of beluga whales in Cook Inlet, Alaska, declined severely in the 1990s, attributed to unrestricted hunting by Alaska Natives [38, 39]. It has not begun to recover despite a regional moratorium on hunting beginning in 2006 and was listed as Endangered under the U.S. Endangered Species Act in 2008. Estimated to comprise 1300 individuals in the early 1970s, it now numbers fewer than 300 individuals and was estimated to be declining at more than 2% each year for the period 2008–2018 [40]. Cook Inlet belugas face a number of possible threats, including contamination, pathogens, noise, habitat degradation, ship-strikes, and declines in available prey. Understanding this population’s barriers to recovery requires a holistic understanding of the population’s structure, ecology, and health. These aims may be aided by an understanding of their microbiota.

In this study, we characterize and compare the epidermal microbiota of two beluga whale populations: the endangered population in Cook Inlet and the nearest neighboring, purportedly healthy population, in Bristol Bay. This is the first study of the microbial communities associated with beluga whales. Our aims are 1) to describe the core epidermal microbial community associated with Alaska beluga whales, thus providing insight into the beluga-specific microbiome, 2) to understand variability in epidermal microbial communities, and factors that may affect that variability, and 3) to examine links between beluga epidermal microbial communities and beluga health that may improve our understanding of population-level health and aid the conservation of endangered populations.

## Methods

### Sample collection

Samples were collected between 2012 and 2018 in Cook Inlet (*n* = 42) and 2008 and 2016 in Bristol Bay (*n* = 44). Most samples from Cook Inlet were collected during biopsy surveys in 2016–2018, with a small number of additional samples from recent strandings (*n* = 5). Biopsy samples were collected from the upper flank near the dorsal ridge, using a remotely deployed biopsy dart to extract a section of skin and blubber tissue approximately 8 mm in diameter and up to 20 mm in length. Samples were transferred to a liquid nitrogen dry shipper immediately following collection and stored at − 80 °C prior to processing. In Bristol Bay, tissue samples were collected from whales that were captured near shore (*n* = 44) following the protocol outlined in Goertz et al. [41]. Individual whales longer than 250 cm, excluding mom-calf pairs, were first followed in an 18 ft. aluminum skiff until they entered shallow water, at which point the individual was corralled using a net held between the first boat and an additional boat. During capture the animal was restrained using a tail rope, hoop nets, slings, and additional staff as needed. Each individual was held for less than 2 h, during which time it underwent a visual health examination and sex determination, measurements, biological sampling, and satellite tag attachment. Skin and blubber biopsies up to 8 mm in diameter and less than 2 cm deep were taken along the flank below the dorsal ridge to closely approximate the location of remote dart biopsies. Once collected, samples were stored on dry ice in the field and later transferred to liquid nitrogen before final storage at − 80 °C prior to processing.

### Microbial sequencing

DNA was extracted from healthy skin tissue (no lesions) from animals in all states of health, so that the proximity of the sample to diseased tissue could be factored out of resulting analyses. Extractions used approximately 2 mm^3^ of skin tissue, taken from the outer epidermal layer whenever possible, and a DNEasy Blood and Tissue Kit (Qiagen). The kit was modified by first homogenizing the tissue, and then replacing the lysis buffer with the following buffer mix: 20 mM Tris HCl pH 8.0, 2 mM EDTA pH 8.0, 1.2% v/v Triton X100 [4]. Hypervariable IV region of the small subunit ribosomal RNA (SSU rRNA) genes of bacteria and archaea were amplified in triplicate for sequencing. Each PCR contained 1 μ l of DNA, 5 μ l of GoTaq 5X Flexi buffer (Promega), 14.75 μ l of H_2_O, 2.5 μ l of a 25 nM MgC l_2_ solution, 0.5 μ l dinucleoside triphosphate (dNTP; Promega), and 0.25 μ l of a 5 units/ μ l GoTaq DNA polymerase solution per sample, along with 200 nM each primer (515FY and 806RB), which included unique forward and reverse barcodes for each sample [42, 43]. Amplification and barcoding occurred in the same PCR step. PCR cycle number was optimized on a per-sample basis, ranging from 35 to 38 cycles. The amplification protocol was 2 min at 95 °C, 35 to 38 cycles of 20 s at 95 °C, 15 s at 55 °C, and 5 min at 72 °C, followed by 10 min at 72 °C. The triplicate PCR products were pooled for each sample and visualized on a 2% agarose gel with 1% 10,000X SyberSafe dye, and bands were excised from the gel and purified using a MinElute gel purification kit (Qiagen). The gel-purified product was quantified using the Qubit assay (Invitrogen), and barcoded products were pooled in equimolar concentrations for 2 × 250 bp MiSeq sequencing (Illumina) at the Georgia Genomics and Bioinformatics Core, University of Georgia. A negative control was included with each extraction batch and PCR amplification run. These negative controls were carried through to gel-visualization, at which point no product was observed. To evaluate the effectiveness of the amplifications and sequencing runs, two mock communities were amplified and included with each sequencing run: 1) an equimolar community, and 2) a staggered ribosomal RNA operon (BEI Resources, NIAID, NIH, Manassas, VA, USA, as part of the Human Microbiome Project: Genomic DNA from Microbial Mock Community B, HM-782D and HM-783D, v5.2 L). A total of 19,404,299 raw reads were generated from 102 samples in two Illumina MiSeq sequencing runs, including 4 mock communities in two sequencing runs, 8 duplicates (identical samples from the same extraction that were independently amplified with unique barcodes), and 4 samples with no associated metadata, which were removed prior to data analysis. Sequences were deposited in the NCBI’s Short Read Archive under accession numbers SAMN15503410 - SAMN15503499.

To test sequencing accuracy, the program dada2 v1.12 [44] inferred 28 sequences in the mock community, 21 of which were matches to the expected reference sequences. The 7 sequences that did not match the reference sequences were compared to the NCBI database using BLAST. The most common similarities were with uncultured bacterial sequences, although one matched *E. coli* and another matched *Rhodobacter sphaeroides*. The non-reference sequences were not found in any of the samples in the dataset, so it is unlikely that these sequences were the result of contamination in the lab.

### Sequence processing

The resulting sequences were processed using the dada2 pipeline, implemented in R v3.6.1 [45]. Due to the high similarity between duplicates, one duplicate from each identical pair was chosen to represent duplicated samples (Supplemental Figure S1). Sequences were first trimmed to remove low-quality regions at the tails by cutting sequences once the Phred score fell below 10. Next, sequences were removed if they had more than two expected errors per sequence, calculated based on the Phred quality score:

$$ EE=\sum \limits_{n=1}^{seqlenth}\left({10}^{\left(-Q/10\right)}\right) $$

Filtered forward and reverse sequences were then dereplicated based on the estimated error rate for each possible nucleotide transition, unique sequences were identified using pseudo-pooling, and paired reads were merged to generate full denoised sequences. Denoised sequences were clustered into amplicon sequence variants (ASVs), and chimeras were removed. ASVs were favored over more traditional OTUs for increased precision, taxonomic resolution, and reproducibility [46]. After quality filtering with dada2, 12,147,026 reads remained. At this stage, samples with fewer than 10,000 reads (*n* = 8) were removed from the dataset (mean reads = 122,163.2 ± 75,922.46). Additionally, the 5 samples from stranded individuals were determined to have significantly different microbial communities compared to non-stranded individuals in Cook Inlet, based on a PERMANOVA of Bray-Curtis dissimilarity metrics [47], and were removed from the dataset prior to further analysis. Taxonomy was assigned using the Silva rRNA sequence database (version 132) [48]. Non-target sequences included 16 ASVs that could not be classified by the Silva database; these were determined by an NCBI search to be cetacean, fungal, algal, or parasite and manually removed from the dataset. Removing eukaryotic sequences caused one additional sample to fall below the minimum threshold of 10,000 reads (sample DL8–8), and it was also removed from the final dataset. Quality pruning of the dataset resulted in a final dataset comprising 72 samples (33 in Cook Inlet, 39 in Bristol Bay).

### Data analysis

Geographic and temporal variability in the core microbial community, as well as variability driven by sex and age, were assessed using a Principal Coordinates Analysis (PCoA) of Bray-Curtis dissimilarity, implemented in the package phyloseq [49], and Permutational Multivariate Analysis of Variance (PERMANOVA) implemented using the package vegan [50]. Observed richness, as well as Shannon and Simpson alpha diversity metrics [51, 52], were also calculated using phyloseq; similarly, beta diversity [53] within each population was estimated using pairwise Bray-Curtis dissimilarity among individuals within that population.

Correlation between epidermal microbial communities and individual health status was tested on a subset of the samples in the study, comprising individuals live-captured for sample collection in Bristol Bay. At the time of capture, qualitative health assessments were performed by a veterinarian from the Alaska SeaLife Center (CECG). Based on these health assessments, animals were designated as either healthy (*n* = 14) or exhibiting evidence of skin disease (*n* = 15). Because many marine pathogens remain undiscovered, we looked for possible pathogenic microbes by identifying ASVs in the same genera as known pathogens [54]. We tested for differences in abundance of these potential pathogens between healthy and diseased individuals using an analysis of differential representation of ASVs, implemented in DESeq2 [55].

Health assessments for Bristol Bay animals also identified individual molt status; using this information we stratified all healthy individuals into two categories based on whether they were molting (*n* = 4) or non-molting (*n* = 10). Epidermal microbial communities of molting and non-molting individuals were compared using alpha diversity indices, estimated using the package phyloseq. We further looked for specific microbes from the entire dataset, as well as specific microbes from the potential pathogen dataset, with differential abundances between the two groups using DESeq2.

## Results

Sequence clustering of the 7,323,872 quality, target SSU rRNA gene sequencing reads (average per sample = 95,115, range of 16,296 - 238,307) from Bristol Bay and Cook Inlet skin microorganisms generated 13,532 ASVs among the 72 samples. The median percent per sample of non-target, eukaryotic reads removed from analysis was 0.028%; 9 individuals had > 1% non-target, eukaryotic reads. A total of 75.5% of target ASVs were classified to family, and 50.55% to genus.

Archaea were represented by 158 ASVs, primarily from the classes Nitrososphaeria and Thermoplasmata. Archaea were found in 52 samples, in both Cook Inlet and Bristol Bay, and comprised an average of 1.4% of the skin microbial communities. One outlier, sampled in Cook Inlet in 2018 (Sample DL18–6, Supplemental Table S1), had a microbial community with 42% archaeal composition. Archaea comprised < 5% of all other samples. The remaining ASVs classified as Bacteria.

### Beluga core epidermal microbial community

Beluga whale microbial communities were dominated by the phyla Bacteroidetes and Proteobacteria, which made up an average of 11 and 51% of individual community composition, respectively. No individual ASVs or classified genera were present in all samples included in the study, but two families were present in all samples: Moraxellaceae (mean 18%) and Burkholderiaceae (mean 5%). Individual traits, including sex and age-class, had a significant but small effect on microbial community composition when all samples were considered (age-class PERMANOVA on Bray-Curtis dissimilarity; *R*^*2*^ = 0.053, *p*-value = 0.017, sex PERMANOVA *R*^*2*^ = 0.02, *p*-value = 0.035).

### Geographic and temporal variability in microbial community

No individual ASVs were present in all individuals in either the Cook Inlet or Bristol Bay population. In Cook Inlet, no classified genera were present in all individuals. However, in Bristol Bay, three genera were present in all individuals: *Acinetobacter, Flavobacterium*, and *Pseudomonas*. Those genera each comprise 1309, 798, and 833 ASVs, respectively, within the Bristol Bay population.

**Fig. 1:**
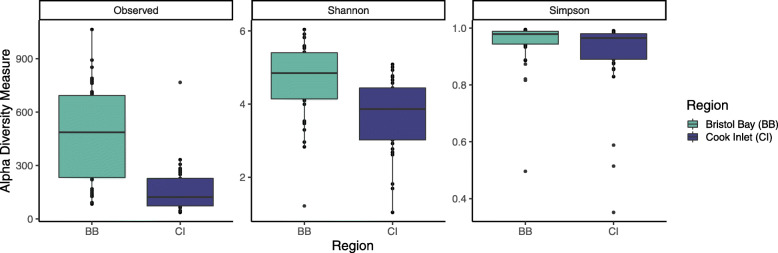
Observed richness (number of ASVs), Shannon, and Simpson indices of alpha diversity among epidermal microbiomes in Bristol Bay (BB) and Cook Inlet (CI) beluga whales

**Fig. 2:**
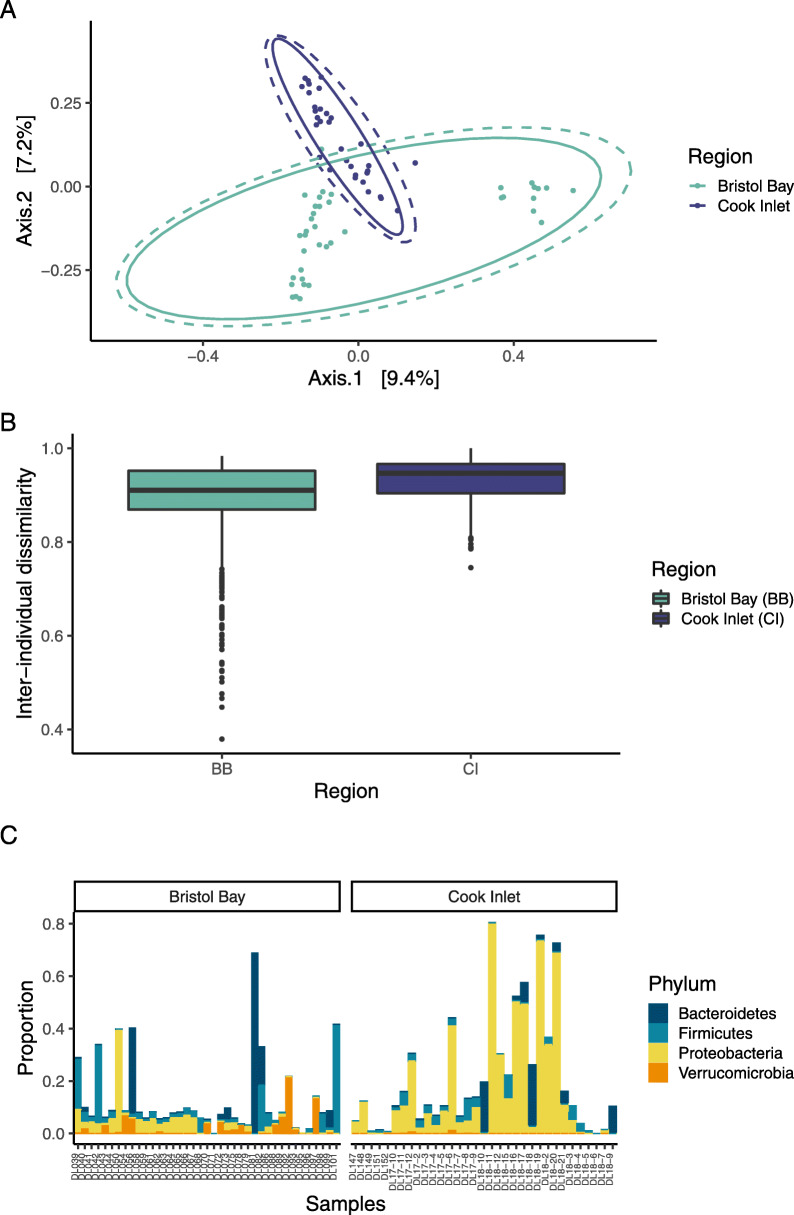
Comparison of epidermal microbial communities in Bristol Bay and Cook Inlet beluga whales. **a** PCoA based on Bray-Curtis dissimilarity comparison of the epidermal microbiota. Multivariate normal and t-distributional data ellipses are shown in dotted and solid lines, respectively. **b** Beta diversity within each regional population, estimated by calculating Bray-Curtis dissimilarity between individual microbial communities within populations. **c** Proportional abundances of top 20 ASVs, organized by phylum, found on individual whales in Bristol Bay and Cook Inlet

Epidermal microbiome alpha diversity differed significantly between the two regions in three major indices: observed richness, Shannon, and Simpson indices (PERMANOVA *R*^*2*^ = 0.28, *p* = 0.001, Fig. 1), with higher diversity in the Bristol Bay population across all three indices. Microbial community composition also differed significantly between regions, based on a Principal Coordinate Analysis (PCoA) of Bray-Curtis dissimilarity (Fig. 2a) and PERMANOVA testing (*R*^*2*^ = 0.055, *p*-value = 0.001). Beta diversity (dispersion) was high within each region, indicating a large degree of dissimilarity in individual microbiomes within each of the two populations, and was significantly higher in Cook Inlet than in Bristol Bay (one-sided Wilcoxon *p*-value < 0.001), with greater variability in beta diversity in Bristol Bay than in Cook Inlet (Fig. 2b). Bacteroidetes and Firmicutes were common on beluga whales from Bristol Bay, while individual communities from Cook Inlet were dominated by Proteobacteria (Fig. 2c).

**Fig. 3:**
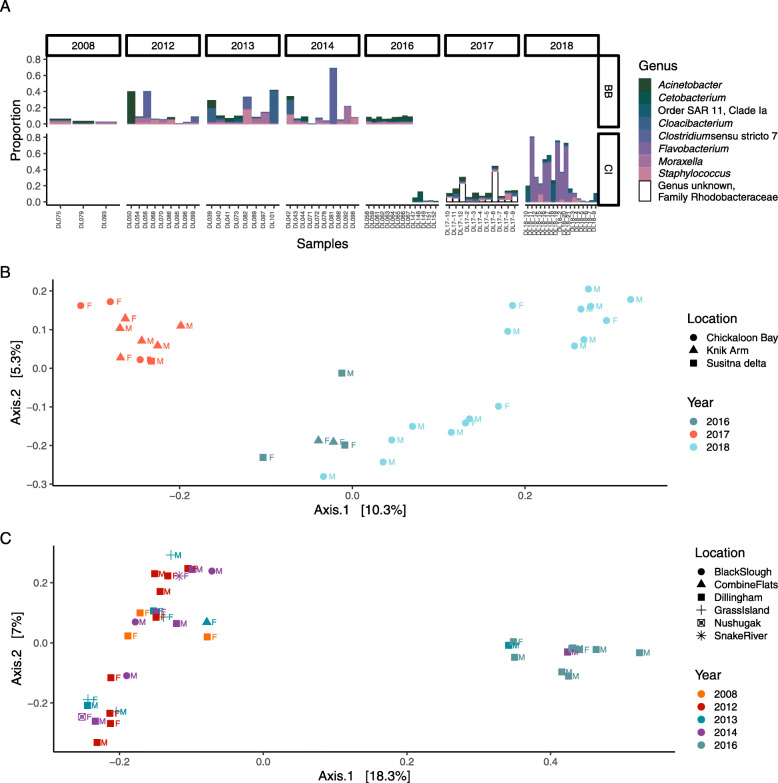
Annual variability within each regional population. **a** Variation in proportional abundance of top 20 ASVs associated with individual whales in Bristol Bay (top row) and Cook Inlet (bottom row), organized by genus. Each bar represents an individual sample, grouped by year along the x-axis. The bottom two plots are PCoA analyses based on Bray-Curtis dissimilarity comparison of epidermal microbiota, with color by year, shape by sampling location, and label by sex, in **b** Bristol Bay and **c** Cook Inlet

Inter-annual variability was apparent among years within both populations (Fig. 3), with high inter-individual variability within years. Bristol Bay skin microbial communities were dominated by Proteobacteria, including genera such as *Acinetobacter* in 2008 and SAR11 clade Ia in 2016, with a shift toward Bacteroidetes (*Flavobacterium* sp.) and Firmicutes (*Staphylococcus* sp. and *Clostridium* sp.) in the interim years. We did not have a sufficiently long time series to look for a similar pattern in the Cook Inlet population, but in that population differences were apparent between 2017 and 2018 at the sub-phylum level within the Proteobacteria. When region and year are considered in the same PERMANOVA, year has a strong effect on variability in epidermal microbial communities (*R*^*2*^ = 0.19, *p* = 0.001), and the geographic effect of region is diminished, i.e. R^2^ falls from 0.055 to 0.039 (*p* = 0.001). Within Cook Inlet, epidermal microbiomes differed significantly among the three locations where samples were collected (PERMANOVA *R*^*2*^ = 0.097, *p*-value = 0.001), as well as among age classes (PERMANOVA *R*^*2*^ = 0.073, *p*-value = 0.036), although these effects may be conflated with annual effects (Fig. 3b). Within Bristol Bay, the magnitude of effect of location was greater than in Cook Inlet among samples in this study, but it was non-significant (PERMANOVA *R*^*2*^ = 0.15, *p*-value = 0.058). The effect of age class in Bristol Bay was similar to that in Cook Inlet (PERMANOVA *R*^*2*^ = 0.069, *p*-value = 0.039). In this population as well, the effects of sampling location, age class, and sampling year may be conflated (Fig. 3c).

Comparing microbial communities to the database of human and animal pathogens developed previously [54] at the genus level revealed 2,313 ASVs associated with pathogenic genera in both populations. The most abundant potentially pathogenic genera across all samples were *Moraxella* and *Acinetobacter*. The composition of pathogenic microbes varied with year (PERMANOVA *R*^*2*^ = 0.29, *p*-value = 0.001), but not between the two regions.

### Epidermal microbial variability and individual health status

**Fig. 4:**
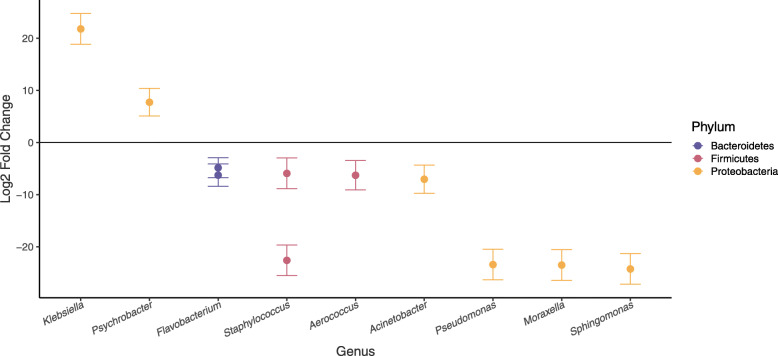
Log2 fold change (i.e. log-of-the-fold-change) in abundance of potentially pathogenic genera that differed significantly between healthy and diseased individuals. The magnitude and direction of change is displayed as the change in abundance of microbes on diseased individuals when compared to healthy individuals (i.e., genera enriched in diseased animals are positive values and those enriched in healthy individuals are negative values)

Epidermal health status was assessed for 29 individuals live captured and released in Bristol Bay. Overall, healthy individuals and those with skin disease did not exhibit significant differences in the proportion of potentially pathogenic ASVs present (t-test *p*-value = 0.14) or composition of potential pathogens (PERMANOVA *R*^*2*^ = 0.027, *p*-value = 0.922). However, testing for differential abundances of each potential pathogen between diseased and healthy individuals revealed 11 genera with significantly different abundances (using a p-value threshold of 0.05) between the two groups (Fig. 4). Of those, two ASVs were more abundant in diseased animals: *Klebsiella* sp. and *Psychrobacter* sp.

**Fig. 5:**
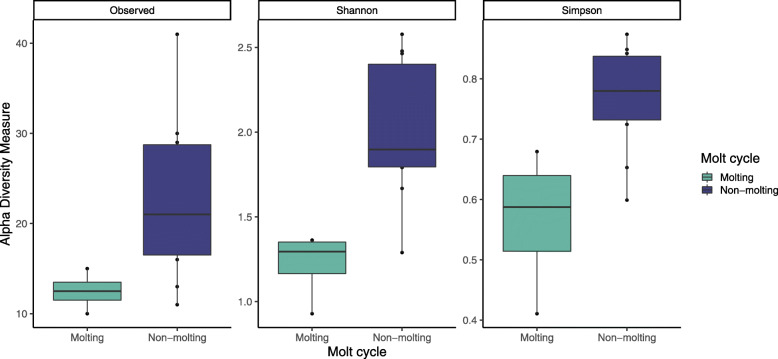
Observed richness (number of ASVs), Shannon, and Simpson indices of alpha diversity among epidermal microbial communities in actively molting and non-molting animals live-captured in Bristol Bay

**Fig. 6:**
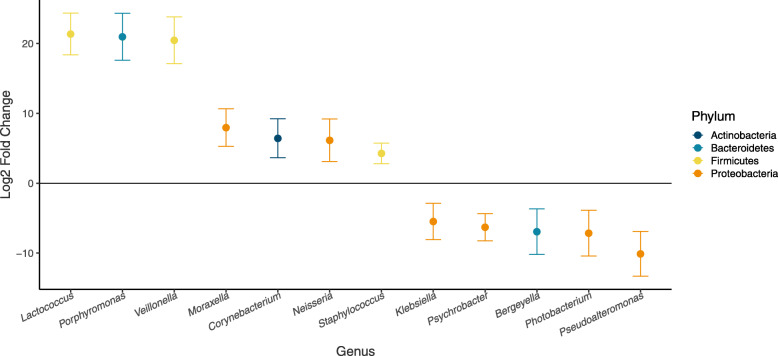
Log-fold-change in abundance of potentially pathogenic genera that differed significantly between molting and non-molting individuals. The magnitude and direction of change is displayed as the change in abundance of microbes on non-molting individuals when compared to molting individuals

Molting and non-molting animals in Bristol Bay exhibited differences in overall microbial community diversity, with a higher diversity of potentially pathogenic ASVs in individuals that were not molting (PERMANOVA *R*^*2*^ = 0.34, *p* = 0.019, Fig. 5). It is important to note the small sample size of molting individuals (*n*= 4), which often decreases precision in distributional means. An analysis of differential abundance of potentially pathogenic microbes between molting and non-molting individuals isolated 12 genera with significantly different abundances (*p* < 0.05) between the two groups (Fig. 6), five of which were more abundant in non-molting individuals.

## Discussion

### No core epidermal microbial community in belugas

Unlike previous studies of cetacean microbiomes [2, 4], the results of this study do not present strong evidence for a species-specific core microbial community in Alaskan beluga whales. No ASVs were found on all whales included in the study. While this may be attributed partially to the long-term isolation of these two populations [56], it was also true that no ASVs were found in all whales within either of the two populations. The variability in epidermal microbiome could be affected by a number of factors (discussed further below), including social group membership, annual or seasonal variability in environmental factors or individual behavior, or preferred habitat within each region.

Individual and population health could also be important drivers of increased microbial diversity and the lack of a core microbiome in this study, as per the Anna Karenina Principle (AKP) in microbiomes, which posits that ‘all healthy microbiota are all alike; each unhealthy microbiome is dysbiotic in its own way’, i.e. that healthy individuals will have similar microbiota while diseased individuals will each deviate from the “healthy microbiome” in a unique way, such that the very presence of disease can increase microbiome beta diversity stochastically [57]. In human microbiomes this principle has been shown to hold true for approximately 50% of diseases, while the remaining diseases exhibited either anti-AKP effects (25%) or effects unrelated to AKP (25%) [58]. In this study, beta diversity was high in both populations, and higher overall in Cook Inlet than in Bristol Bay. However, at a population level it is difficult to interpret beta diversity as an indicator of disease, as individual diseases have a nearly equal likelihood of correlation with increased beta diversity vs. decreased or equal beta diversity in individual host microbiomes; thus the composition of diseases in a population may have more effect on beta diversity than the abundance of diseases in that population.

Seawater control samples were not available as part of this study; therefore, it is important to consider that some of the documented microbiome variability may be contributed by seawater microbiota. Previous studies indicate that the contribution from seawater microbes is likely limited, as marine mammals harbor microbial communities that are distinct from their surrounding environments [2, 4, 9]. However, the collection of seawater control samples as part of future studies will allow for the determination of whether and how much variability in the epidermal microbiome of belugas is affected by seawater-associated microbes.

Only 50% of ASVs were classified to genera, highlighting our paucity of knowledge when it comes to marine mammal-hosted microbial communities, and the need for continued study of the relationships between these animals and their microbiota. Proteobacteria, Bacteroides, and Firmicutes were the most common phyla found in this study; of these, the first two are common in both cetacean and seawater microbiomes, and Firmicutes has also been documented in marine mammal oral and rectal microbiomes [2]. The lowest taxonomic level found to be common across all samples was that of family: Moraxellaceae and Burkholderiaceae were present on all samples in both populations in this study. Moraxellaceae is a family of gammaproteobacteria, including known psychrotrophic species that would be well-suited to the polar habitat of beluga whales [59, 60]. The family includes both pathogenic and commensal species, but the majority of ASVs from the family Moraxellaceae that were documented in this study remain unidentified and unstudied, therefore it is unknown whether they are pathogenic. The second family occurring across all samples, Burkholderiaceae, is a broadly diverse family represented by over 50 genera in this study. This family includes multiple examples of genera that tolerate or thrive in extreme cold environments, including *Janthinobacterium* [61, 62] and the psychrophile *Polaromonas* [62, 63]. The family also includes a number of genera more commonly associated with wastewater or contaminants, such as *Extensimonas* [64] and *Sphaerotilus* [65–67], which are found in industrial wastewater and activated sludge, and were both documented on a small number of individuals from each population. In Bristol Bay, these animals were sampled near Dillingham; in Cook Inlet they were sampled in Eagle River in Knick Arm, and in Chickaloon Bay. While it is possible that these bacteria are part of the core epidermal microbial community, they are more likely indicative of the whales’ interactions with contaminated nearshore waters in both regions. The presence of these genera in the epidermal microbiome should not be overlooked; future studies should consider the extent and concurrence of wastewater microbiota on beluga whales and their preferred habitats, as well as the potential effects to beluga health of these microbiota or associated contaminants.

While no genera were common to all sampled individuals in Cook Inlet, three genera were found on all individuals in Bristol Bay*: Acinetobacter, Flavobacterium,* and *Pseudomonas. Pseudomonas* has previously been documented among delphinids and phocids, and may be a cosmopolitan-type associate of marine mammals [5]. The genus contains species with a wide range of metabolic diversity and niche space, and includes several known pathogens of humans, non-human animals, and plants [68]. Further research is required to determine the role of this genus in marine mammal microbiota, and whether the organisms in this genera have a similar function across all host relationships. *Acinetobacter* has also been documented in humpback, grey, and pilot whale skins [69, 70], is common in seawater, and is able to live in a wide range of temperatures and environmental conditions [71]. Coral-associated species of *Acinetobacter* have been shown to produce chemicals that inhibit biofouling [72]. However, this genus also harbors potentially pathogenic species [54]; the abundance of this genera warrants further research into to functional roles of beluga-associated species within the *Acinetobacter*, and whether those species are harmful or beneficial. Finally, *Flavobacterium* has been previously identified in the core epidermal microbiome of humpback whales [69]; the genus also includes several pathogens of freshwater and saltwater fishes [73, 74].

It is worth noting that Archaea, which have never before been documented in marine mammal microbiomes despite some use of primers that could amplify members of this domain, were present in nearly 70% of the samples in this study, although generally in low proportions. The paucity of information on Archaea makes it difficult to hypothesize the cause for the outlier with 42% archaeal composition, which was an adult male sampled in Cook Inlet in 2018; however, there is no indication that it was caused by contamination. The domain Archaea is probably most well-known for comprising extremophilic species, but have been found to be part of the microbiota of many organisms [75–77], and are especially numerous in marine environments [78, 79]. They are an important part of the epidermal microbiota of humans [80], in similar proportions to that found here on beluga whales [81]. In humans, they are thought to be important to nitrogen turnover and skin health [81]; they may have a similar role in the epidermal microbiota of beluga whales and other marine cetaceans. There are no known pathogenic or parasitic Archaea; they are more often mutualist or commensals.

### Geographic and temporal variability in epidermal microbiome

Spatial and temporal variability are common in host-associated microbiomes [69, 82–84]. The majority of microbiome research is still focused on humans and humanized model systems, and that research shows geographic and temporal variability in microbiomes even among healthy adults [85]. Characterizing this type of variability in a species, which is likely at least partially driven by environmental factors, will improve our understanding of a species’ interactions with its ecosystem. Further, it allows scientists to isolate and understand microbiome variability that may be related to changes in individual or population-level health, providing key baseline data for developing monitoring tools critical for evaluating individual and population-level responses to environmental change.

Beluga whale microbiotas differed both spatially and temporally, indicating a strong environmental effect on microbial community composition that should be further investigated. However, these results should be interpreted with caution due to minimal annual overlap in sampling between the two geographic populations. Future research aiming to collect samples from both regions in the same year and season would greatly enhance our ability to detect temporal and regional effects on microbiome variability.

Alpha diversity was higher in Bristol Bay than in Cook Inlet, a phenomenon that could be driven in part by endogenous population factors, such as the greater long-term abundance and density of beluga whales in Bristol Bay. When individual beluga whales are considered as habitat space for microbial populations, the existence of less habitat space in Cook Inlet may lead to increased local extinction rates, thus decreasing microbial diversity. Similarly, the higher density of beluga whales in Bristol Bay may lead to increased opportunities for interactions among microbial communities, thus increasing microbial transfer and decreasing variability among individuals. This is reflected in the beta diversity metric, which was lower in Bristol Bay than in Cook Inlet. However, beta diversity was generally high within both populations, indicating high variability among individuals - some of this is likely accounted for by other patterns detected in this study, such as annual variability within populations, molt stage, and individual health status.

Bray-Curtis dissimilarity metrics indicate strong divergence between the two populations, which could be driven by a number of factors. Genetic evidence suggests the Cook Inlet population is demographically isolated from Bristol Bay and other populations in the Bering Sea [56]. Migration between the two populations is minimal over recent (ecological) timescales, according to a Bayesian analysis of eight nuclear loci that estimate a recent migration rate from Bristol Bay to Cook Inlet of 0.004 (95% confidence interval: 0–0.03) [56]. This value approximates one individual (95% confidence interval: 0–8 individuals) over the last two generations, or roughly 58 years, based on a population of 300 individuals and a generation time of 29 years [86], although this estimate is rough due to uncertainty in all three estimates (migration rate, generation time, and population abundance), which compound in this calculation. Similarly, it is estimated from population genetic data that in the last ~ 60 years the Bristol Bay population may have received six immigrants from Cook Inlet (95% confidence interval 0–22 individuals) based on a population of 2000 individuals [35]. With connectivity this low between the two populations, their microbial communities are almost entirely isolated, which facilitates both random and adaptive divergence between them. The differences between regions could also be explained by environmental differences between habitats, including natural differences such as oceanographic or benthic properties of the two bodies of water, as well as anthropogenic differences such as nutrient and contaminant loading.

Observed annual variability within populations could also be attributed to a number of potential drivers, including endogenous factors, annual changes in environmental conditions, or anthropogenic disturbance. In this study, we documented an effect of age on microbial community composition both between the two regions and within the Cook Inlet population. Sex was also found to have a small-but-significant effect, similar to age, both of which may reflect behavioral or endogenous differences among groups (e.g. group associations, hormone levels and/or inherent biochemical differences, diet preferences). Males and females, for example, are thought to prefer different habitats [87]. If that is the case, it is possible that sex-based habitat preferences may be confounded with that of socially driven habitat preferences. Previous studies have found that water temperature can be an important driver in epidermal microbiome composition [69], and may have an effect here as well. To determine the relative contribution of endogenous and environmental factors to microbial community variability, it will be important to conduct studies that vary one factor while sampling randomly across the others, as it is not feasible in wild populations to hold the other factors constant.

Intra-population variability may also be socially driven, either by affecting the likelihood of sampling an individual in a given year, or by affecting the individual’s preferred habitat and, by proxy, its epidermal microbiome. Within Cook Inlet we observed strong differences in epidermal microbiome associated with sampling location. Individuals migrate around the inlet throughout the year, so it is unknown whether this correlation represents rapid turnover of epidermal microbiome driven by local environmental conditions, or sub-population structure driven by differences in ecological behaviors. If the former, it would be the first documentation of rapid turnover in cetacean epidermal microbiome in response to local environmental conditions, and would suggest strong variability in water quality and habitat characteristics throughout Cook Inlet. The latter possibility is more common. Ecological behaviors such as migration routes and habitat use have been shown to vary by social group membership in this species [88, 89], which could affect both annual and spatial variability within Cook Inlet. Further, this species is thought to have a highly complex, stable social structure [17, 90, 91], which may affect individual microbial communities in a number of ways, both directly (e.g. influencing patterns of transmission among individuals) and indirectly (e.g. driving habitat use and therefore exposure to specific microbes). The large range in beta diversity metrics (0.38–1) supports the hypothesis of socially driven transmission of epidermal microbiomes. It is possible that those individuals with more similar microbiomes are closely related or associate more often. Future work exploring correlations between individual microbiota and kinship, or measures of social affiliation, may indicate an important link between social structure and microbial communities, and ultimately help us understand the pathways of disease transmission in this species.

### Epidermal microbial variability and individual health status

One of the goals in understanding variability in the epidermal microbial communities of cetaceans is to develop a minimally-invasive, rapid method to determine the health status of individuals [9]. Among humans, where the field of host microbiome research has been concentrated for the last decade, emerging results indicate the potential power of this research as a health indicator in more elusive species. Microbiome indicators of health outperform genome-wide association studies by nearly 20% [92], and certain microbial taxonomic groups have been linked with an increased probability of mortality [93]. In some cases, specific taxa have been linked with common diseases, such as autism [94], allergies [95], and obesity [96, 97].

Examining the differential abundance of potentially pathogenic species revealed 11 ASVs that may be key indicators of individual health status. The two ASVs that were more abundant in diseased animals were classified as *Psychrobacter* and *Klebsiella*. The first, *Psychrobacter* sp., was nearly 10 times more abundant in diseased animals than in their healthy counterparts. *Psychrobacter* are gram-negative psychrophiles often found in cold marine environments [98–100], whose endotoxins have been shown to cause an inflammatory response in macrophages, and which are known to cause human infections including endocarditis and peritonitis [101]. Yet *Psychrobacter* are abundantly associated with the skin microbiome of healthy humpback whales [9, 69], thus indicating that much is still unknown about how these microbes interact with their hosts. The second, *Klebsiella* sp., was > 20 times more abundant in diseased animals. *Klebsiella* display a wide range of specialized adaptations that allow them to be present in nearly all habitats and niches [102]. They are opportunistic pathogens in humans, with species known to cause a variety of internal diseases and soft-tissue infections [103]. Recent research outside of humans indicates *Klebsiella* may comprise species that infect non-human animals, as well [104]. Identifying and studying the functional role of the particular microbes living on beluga skin could be an important step toward understanding beluga health, but further work is needed to understand their functional roles and test their reliability as indicators of disease in an individual or population.

Nine additional potentially pathogenic ASVs in seven genera were less abundant in diseased animals, which was not expected and may indicate that, despite their close relationship to known pathogens, the ASVs represented by these genera are commensal and may be important to maintaining healthy skin. Additional research should be directed toward isolating the ASVs identified here in order to study their taxonomy and function, and to determine their role in beluga health. It is important to note that the sample size used for this analysis was moderate (*n*= 29), and samples were collected in a number of years. As shown above, variability among years was high, therefore additional variability due to sample size may either diminish or magnify differences in abundance of pathogenic microbes. In order to control for outside effects on microbial variability when testing the role of these genera in beluga health, it will be necessary to collect a large, random sample of epidermal tissue from healthy and sick individuals from the same population and year.

Despite a relatively small sample size of molting vs. non-molting individuals we found differences in microbial community diversity (Fig. 5), as well as 12 potentially pathogenic microbes that differed in abundance between molting and non-molting individuals, five of which were more prevalent in non-molting individuals than those that were molting. This finding suggests a possible role of molting in epidermal maintenance; the general reduction in bacterial diversity likely helps to remove harmful bacteria to maintain skin health in beluga whale populations, although the same limitations of the above analysis are true here, and a directed study of molting vs. non-molting individuals would be necessary to confirm the results reported here. The annual molt of beluga whales has long been thought to be important to maintaining skin health by removing damaged or dead epidermal tissue that may be colonized by abundant microbiota [20]. As this is the first study of epidermal microbiomes in a polar cetacean species that undergoes an annual molt, it is worth noting that the lack of a core microbiome may be related to the large differences found in the microbiota of molting vs. non-molting animals, and that future studies may compare microbiomes among groups stratified by molt stage in order to better understand the effect of molting on beluga epidermal microbiota.

## Conclusion

This study represents an important first step toward understanding epidermal microbial communities in beluga whales, as well as their role in beluga health. Describing natural variability within the healthy Bristol Bay population, and comparing that with the endangered Cook Inlet population, may serve as a baseline for comparisons among healthy and affected populations. Additionally, isolating healthy and diseased individuals and describing differences in their epidermal microbiomes allows us to begin to develop indices for rapid, less-invasive health assessments of these animals, using tissue biopsies or swabbing rather than live captures. We found no support for the presence of a core microbial community in beluga whales, unlike other species studied to date, and found that within each population, microbial communities shifted annually; however, with only two populations compared it is possible that there is a common biota among most populations and one or both of these are anomalous. Intra-population variability could not be fully explained by this study; it is possible that it may be further explained by individual differences in ecological behaviors or social group membership. Within Bristol Bay, we identified individual microbes that differ in abundance between healthy and diseased animals, and which may be used to develop health metrics that can be applied to beluga whales in the endangered Cook Inlet population, thus potentially providing managers some measure of insight into the question of why this population has not recovered in the nearly 15 years since the 2006 hunting moratorium.

## Supplementary information

**Additional file 1: Supplemental Figure S1.**. PCoA based on Bray-Curtis dissimilarity comparison of the epidermal microbiota of duplicate samples included in this study.

**Additional file 2: Supplemental Table S1.**. Sample metadata for all samples included in this study (i.e. all samples that passed quality control analyses), including sampling location and population, year, and NCBI Genbank Accession number, among other data.

## Data Availability

All data used in this study are publicly available in the NCBI database, stored under accession numbers SAMN15503410 - SAMN15503499.

## Abbreviations

AFSC/MML: Alaska Fisheries Science Center/Marine Mammal Laboratory
AKP: Anna Karenina Principle
ASV: Amplicon sequence variant
BLAST: Basic Local Alignment Search Tool
DNA: Deoxyribonucleic acid
dNTP: Deoxynucleoside triphosphate
EDTA: Ethylenediaminetetraacetic acid
NCBI: National Center for Biotechnology Information
NOAA: National Oceanic and Atmospheric Administration
PCoA: Principal Coordinates Analysis
PCR: Polymerase chain reaction
PERMANOVA: Permutational Multivariate Analysis of Variance
SSU rRNA: Small subunit ribosomal ribonucleic acid
SWFSC: Southwest Fisheries Science Center
Tris HCl: Tris hydochloride
WHOI: Woods Hole Oceanographic Institution

## References

[1] DeLong EF, Taylor LT, Marsh TL, Preston CM (1999). Visualization and enumeration of marine planktonic archaea and bacteria by using polyribonucleotide probes and fluorescent in situ hybridization. Appl Environ Microbiol.

[2] Bik EM, Costello EK, Switzer AD, Callahan BJ, Holmes SP, Wells RS (2016). Marine mammals harbor unique microbiotas shaped by and yet distinct from the sea. Nat Commun.

[3] Vendl C, Ferrari BC, Thomas T, Slavich E, Zhang E, Nelson T, et al. Interannual comparison of core taxa and community composition of the blow microbiota from East Australian humpback whales. FEMS Microbiol Ecol. 2019;95:1-8. 10.1093/femsec/fiz102 31260051

[4] Apprill A, Mooney TA, Lyman E, Stimpert AK, Rappé MS (2011). Humpback whales harbour a combination of specific and variable skin bacteria. Environ Microbiol Rep.

[5] Apprill A, Miller CA, Van Cise AM, U’Ren JM, Leslie MS, Weber L (2020). Marine mammal skin microbiotas are influenced by host phylogeny. R Soc Open Sci.

[6] Wahl M, Goecke F, Labes A, Dobretsov S, Weinberger F (2012). The second skin: ecological role of epibiotic biofilms on marine organisms. Front Microbiol.

[7] Marzinelli EM, Campbell AH, Zozaya Valdes E, Vergés A, Nielsen S, Wernberg T (2015). Continental-scale variation in seaweed host-associated bacterial communities is a function of host condition, not geography. Environ Microbiol.

[8] Nelson TM, Apprill A, Mann J, Rogers TL, Brown MV (2015). The marine mammal microbiome: current knowledge and future directions. Microbiol Australia.

[9] Apprill A, Robbins J, Eren AM, Pack AA, Reveillaud J, Mattila D (2014). Humpback whale populations share a core skin bacterial community: towards a health index for marine mammals?. PLoS One.

[10] Gilbert JA, Quinn RA, Debelius J, Xu ZZ, Morton J, Garg N (2016). Microbiome-wide association studies link dynamic microbial consortia to disease. Nature..

[11] Harrison R, Thurley K, Harrison RJ (1974). Structure of the epidermis in *Tursiops*, *Delphinus* and *Phocoena*. Functional anatomy of marine mammals.

[12] Grice EA, Kong HH, Conlan S, Deming CB, Davis J, Young AC (2009). Topographical and temporal diversity of the human skin microbiome. Science..

[13] Grice EA, Snitkin ES, Yockey LJ, Bermudez DM, Liechty KW, Segre JA (2010). Longitudinal shift in diabetic wound microbiota correlates with prolonged skin defense response. Proc Natl Acad Sci U S A.

[14] Kong HH (2011). Skin microbiome: genomics-based insights into the diversity and role of skin microbes. Trends Mol Med.

[15] Hobbs RC, Waite JM, Rugh DJ (2000). Beluga, *Delphinapterus leucas*, group sizes in cook inlet, Alaska, based on observer counts and aerial video. Mar Fish Rev.

[16] Shelden KE, Goetz KT, Rugh DJ, Calkins DG, Mahoney BA, Hobbs RC (2015). Spatio-temporal changes in Beluga whale, *Delphinapterus leucas*, distribution: results from aerial surveys (1977-2014), opportunistic sightings (1975-2014), and satellite tagging (1999-2003) in cook inlet, Alaska. Mar Fisheries Rev.

[17] O’Corry-Crowe G, Suydam R, Quakenbush L, Smith TG, Lydersen C, Kovacs KM (2020). Group structure and kinship in beluga whale societies. Sci Rep.

[18] Hobbs RC, Reeves RR, Prewitt JS, Desportes G, Breton-Honeyman K, Christensen T (2019). Global review of the conservation status of monodontid stocks. Mar Fish Rev.

[19] Quakenbush LT, Suydam RS, Bryan AL, Lowry LF, Frost KJ, Mahoney BA (2015). Diet of beluga whales, *Delphinapterus leucas*, in Alaska from stomach contents, March–November. Mar Fisheries Rev.

[20] Aubin DJS, Smith TG, Geraci JR (1990). Seasonal epidermal molt in beluga whales, *Delphinapterus leucas*. Can J Zool.

[21] Boily P (1995). Theoretical heat flux in water and habitat selection of phocid seals and beluga whales during the annual molt. J Theor Biol.

[22] Chernova OF, Shpak OV, Kiladze AB, Azarova VS, Rozhnov VV (2016). Summer molting of bowhead whales *Balaena mysticetus* Linnaeus, 1758, of the Okhotsk Sea population. Dokl Biol Sci.

[23] Pitman RL, Durban JW, Joyce T, Fearnbach H, Panigada S, Lauriano G (2020). Skin in the game: epidermal molt as a driver of long-distance migration in whales. Marine Mammal Sci.

[24] Daniel RG, Jemison LA, Pendleton GW, Crowley SM (2003). Molting phenology of harbor seals on Tugidak Island, Alaska. Marine Mammal Sci.

[25] Hobbs RC, Laidre KL, Vos DJ, Mahoney BA, Eagleton M (2005). Movements and area use of belugas, *Delphinapterus leucas*, in a subarctic Alaskan estuary. Arctic..

[26] Citta JJ, Quakenbush LT, Frost KJ, Lowry L, Hobbs RC, Aderman H (2016). Movements of beluga whales (*Delphinapterus leucas*) in Bristol Bay, Alaska. Mar Mammal Sci.

[27] Moore SE, Shelden KEW, Litzky LK, Mahoney BA, Rugh DJ (2000). Beluga, *Delphinapterus leucas*, habitat associations in cook inlet, Alaska. Mar Fisheries Rev.

[28] Goetz KT, Montgomery RA, Ver Hoef JMV, Hobbs RC, Johnson DS (2012). Identifying essential summer habitat of the endangered beluga whale *Delphinapterus leucas* in cook inlet, Alaska. Endangered Species Res.

[29] Goetz KT, Rugh DJ, Read AJ, Hobbs RC (2007). Habitat use in a marine ecosystem: Beluga whales *Delphinapterus leucas* in cook inlet, Alaska. Mar Ecol Prog Ser.

[30] Castellote M, Thayre B, Mahoney M, Mondragon J, Lammers MO, Small RJ (2018). Anthropogenic noise and the endangered cook inlet beluga whale, *Delphinapterus leucas*: acoustic considerations for management. Mar Fish Rev.

[31] National Marine Fisheries Service (2016). Recovery plan for the Cook Inlet beluga whale (*Delphinapterus leucas*)..

[32] Norman SA, Hobbs RC, Wuertz S, Melli A, Beckett LA, Chouicha N (2013). Fecal pathogen pollution: sources and patterns in water and sediment samples from the upper cook inlet, Alaska ecosystem. Environ Sci: Process Impacts.

[33] Norman SA, Hobbs RC, Goertz CEC, Burek-Huntington KA, Shelden KEW, Smith WA (2016). Potential natural and anthropogenic impediments to the conservation and recovery of cook inlet beluga whales, *Delphinapterus leucas*. Mar Fisheries Rev.

[34] Carter BTG, Nielsen EA (2011). Exploring ecological changes in cook inlet beluga whale habitat though traditional and local ecological knowledge of contributing factors for population decline. Mar Policy.

[35] Citta JJ, O’Corry-Crowe G, Quakenbush LT, Bryan AL, Ferrer T, Olson MJ (2018). Assessing the abundance of Bristol Bay belugas with genetic mark-recapture methods. Mar Mammal Sci..

[36] Lowry LF, Citta JJ, O’corry-Crowe G, Quakenbush LT, Frost KJ, Suydam R (2019). Distribution, abundance, harvest, and status of Western Alaska beluga whale, *Delphinapterus leucas*, stocks. Mar Fish Rev.

[37] Citta JJ, Frost KJ, Quakenbush L, Citta J (2019). Aerial surveys of Bristol Bay beluga whales, *Delphinapterus leucas*, in 2016. Mar Fish Rev.

[38] Mahoney BA, Shelden KEW (2000). Harvest history of belugas, *Delphinapterus leucas*, in cook inlet, Alaska. Mar Fisheries Rev.

[39] Hobbs RC, Shelden KEW, Rugh DJ, Sims CL, Waite JM (2015). Estimated abundance and trend in aerial counts of beluga whales, *Delphinapterus leucas*, in cook inlet, Alaska, 1994-2012. Mar Fish Rev.

[40] Sheldon K, Wade P (2019). Aerial surveys, distribution, abundance, and trend of belugas (*Delphinapterus leucas*) in Cook Inlet, Alaska, June 2018.

[41] CEC G, Burek-Huntington K, Royer K, Quakenbush L, Clauss T, Hobbs R (2019). Comparing progesterone in blubber and serum to assess pregnancy in wild beluga whales (*Delphinapterus leucas*). Conserv Physiol.

[42] Apprill A, McNally S, Parsons R, Weber L (2015). Minor revision to V4 region SSU rRNA 806R gene primer greatly increases detection of SAR11 bacterioplankton. Aquat Microb Ecol.

[43] Parada AE, Needham DM, Fuhrman JA (2016). Every base matters: assessing small subunit rRNA primers for marine microbiomes with mock communities, time series and global field samples. Environ Microbiol.

[44] Callahan BJ, McMurdie PJ, Rosen MJ, Han AW, Johnson AJA, Holmes SP (2016). DADA2: high-resolution sample inference from Illumina amplicon data. Nat Methods.

[45] R Core Team (2016). R: A Language and Environment for Statistical Computing..

[46] Callahan BJ, McMurdie PJ, Holmes SP (2017). Exact sequence variants should replace operational taxonomic units in marker-gene data analysis. ISME J.

[47] Bray JR, Curtis JT (1957). An ordination of the upland forest communities of southern Wisconsin. Ecol Monogr.

[48] Quast C, Pruesse E, Yilmaz P, Gerken J, Schweer T, Yarza P, et al. The SILVA ribosomal RNA gene database project: improved data processing and web-based tools. Nucleic Acids Res. 2013;41. 10.1093/nar/gks1219. 10.1093/nar/gks1219 PMC3531112 23193283

[49] McMurdie PJ, Holmes S (2013). phyloseq: An R package for reproducible interactive analysis and graphics of microbiome census data. PLoS ONE.

[50] Oksanen J, Blanchet FG, Friendly M, Kindt R, Legendre P, McGlinn D (2019). Vegan: Community Ecology Package..

[51] Simpson EH (1949). Measurement of diversity. Nature..

[52] Shannon CE, Weaver W (1964). The mathematical theory of communication.

[53] Whittaker RH (1960). Vegetation of the Siskiyou Mountains, Oregon and California. Ecol Monogr.

[54] Apprill A, Miller CA, Moore MJ, Durban JW, Fearnbach H, Barrett-Lennard LG (2017). Extensive core microbiome in drone-captured whale blow supports a framework for health monitoring. mSystems.

[55] Love MI, Huber W, Anders S (2014). Moderated estimation of fold change and dispersion for RNA-seq data with DESeq2. Genome Biol.

[56] O’Corry-Crowe G, Suydam R, Quakenbush L, Potgieter B, Harwood L, Litovka D (2018). Migratory culture, population structure and stock identity in North Pacific beluga whales (*Delphinapterus leucas*). PLoS One.

[57] Zaneveld JR, McMinds R, Thurber RV (2017). Stress and stability: Applying the Anna Karenina principle to animal microbiomes. Nat Microbiol.

[58] Ma Z (2020). Testing the Anna Karenina Principle in human microbiome-associated diseases. iScience.

[59] Mangano S, Michaud L, Caruso C, Lo GA (2014). Metal and antibiotic resistance in psychrotrophic bacteria associated with the Antarctic sponge *Hemigellius pilosus* (Kirkpatrick, 1907). Polar Biol.

[60] Wu G, Wu G, Zhan T, Shao Z, Liu Z (2013). Characterization of a cold-adapted and salt-tolerant esterase from a psychrotrophic bacterium *Psychrobacter pacificensis*. Extremophiles..

[61] Ley JD, Segers P, Gillis M (1978). Intra- and intergeneric similarities of *Chromobacterium* and *Janthinobacterium* ribosomal ribonucleic acid cistrons. Int J Syst Bacteriol.

[62] Koo H, Strope BM, Kim EH, Shabani AM, Kumar R, Crowley MR, et al. Draft Genome Sequence of *Janthinobacterium sp.* Ant5-2-1, Isolated from Proglacial Lake Podprudnoye in the Schirmacher Oasis of East Antarctica. Genome Announc. 2016;4:1-2. 10.1128/genomeA.01600-15 PMC4722270 26798103

[63] Irgens RL, Gosink JJ, Staley JT (1996). *Polaromonas vacuolata* gen. nov., sp. nov., a psychrophilic, marine, gas vacuolate bacterium from Antarctica. Int J Syst Bacteriol.

[64] Zhang WY, Fang MX, Zhang WW, Xiao C, Zhang XQ, Yu ZP (2013). *Extensimonas vulgaris* gen. nov., sp. nov., a member of the family Comamonadaceae. Int J Syst Evol Microbiol.

[65] Stokes JL (1954). Studies on the filamentous sheathed iron bacterium *Sphaerotilus natans*. J Bacteriol.

[66] Van Veen WL, Mulder EG, Deinema MH (1978). The *Sphaerotilus-Leptothrix* group of bacteria. Microbiol Rev.

[67] Eikelboom DH (1975). Filamentous organisms observed in activated sludge. Water Res.

[68] Euzéby JP (1997). List of bacterial names with standing in nomenclature: a folder available on the internet. Int J Syst Bacteriol.

[69] Bierlich KC, Miller C, Deforce E, Friedlaender AS (2018). Temporal and regional variability in the skin microbiome of humpback whales along the Western Antarctic peninsula. Appl Environ Microbiol.

[70] Vendl C, Slavich E, Nelson T, Acevedo-Whitehouse K, Montgomery K, Ferrari B (2020). Does sociality drive diversity and composition of airway microbiota in cetaceans?. Environ Microbiol Rep.

[71] Doughari HJ, Ndakidemi PA, Human IS, Benade S (2011). The ecology, biology and pathogenesis of *Acinetobacter spp*.: an overview. Microbes Environ.

[72] Olguin-Uribe G, Abou-Mansour E, Boulander A, Débard H, Francisco C, Combaut G (1997). 6-bromoindole-3-carbaldehyde, from an *Acinetobacter Sp*. bacterium associated with the ascidian *Stomozoa murrayi*. J Chem Ecol.

[73] Starliper CE (2011). Bacterial coldwater disease of fishes caused by *Flavobacterium psychrophilum*. J Adv Res.

[74] Declercq AM, Haesebrouck F, Van Den Broeck W, Bossier P, Decostere A (2013). Columnaris disease in fish: a review with emphasis on bacterium-host interactions. Vet Res.

[75] Wegley L, Yu Y, Breitbart M, Casas V, Kline DI, Rohwer F (2004). Coral-associated archaea. Mar Ecol Prog Ser.

[76] Radax R, Hoffmann F, Rapp HT, Leininger S, Schleper C (2012). Ammonia-oxidizing archaea as main drivers of nitrification in cold-water sponges. Environ Microbiol.

[77] Dridi B, Raoult D, Drancourt M (2011). Archaea as emerging organisms in complex human microbiomes. Anaerobe..

[78] Delong EF (1992). Archaea in coastal marine environments. Proc Natl Acad Sci.

[79] Massana R, Murray AE, Preston CM, DeLong EF (1997). Vertical distribution and phylogenetic characterization of marine planktonic archaea in the Santa Barbara Channel. Appl Environ Microbiol.

[80] Bang C, Schmitz RA (2015). Archaea associated with human surfaces: not to be underestimated. FEMS Microbiol Rev.

[81] Probst AJ, Auerbach AK, Moissl-Eichinger C (2013). Archaea on human skin. PLoS One.

[82] Parfrey LW, Knight R (2012). Spatial and temporal variability of the human microbiota. Clin Microbiol Infect.

[83] Moisander PH, Sexton AD, Daley MC (2015). Stable associations masked by temporal variability in the marine copepod microbiome. PLoS One.

[84] Chiarello M, Auguet JC, Bettarel Y, Bouvier C, Claverie T, Graham NAJ (2018). Skin microbiome of coral reef fish is highly variable and driven by host phylogeny and diet. Microbiome..

[85] Gerber GK (2014). The dynamic microbiome. FEBS Lett.

[86] Lowry L, Hobbs R, O’Corry-Crowe G (2019). *Delphinapterus leucas* (Cook Inlet subpopulation). The IUCN Red List of Threatened Species 2019: eT61442A50384653.

[87] Suydam RS, Lowry LF, Frost KJ (2005). Distribution and movements of beluga whales from the eastern Chukchi Sea stock during summer and early autumn. OCS Study Report MMS.

[88] Colbeck GJ, Duchesne P, Postma LD, Lesage V, Hammill MO, Turgeon J (2012). Groups of related belugas (*Delphinapterus leucas*) travel together during their seasonal migrations in and around Hudson Bay. Proc R Soc B Biol Sci.

[89] Turgeon J, Duchesne P, Colbeck GJ, Postma LD, Hammill MO (2012). Spatiotemporal segregation among summer stocks of beluga (*Delphinapterus leucas*) despite nuclear gene flow: implication for the endangered belugas in eastern Hudson Bay (Canada). Conserv Genet.

[90] O’Corry-Crowe G, Perrin WF, Wursig B, JGM T (2002). Beluga whales. Encyclopedia of marine mammals.

[91] Smith TG, Hammill M, Martin A, Born E, Dietz R, Reeves R (1994). Herd composition and behaviour of white whales (*Delphinapterus leucas*) in two Canadian arctic estuaries. Studies of white whales (*Delphinapterus leucas*) and narwhals (*Monodon monoceros*) in Greenland and adjacent waters.

[92] Tierney BT, He Y, Church GM, Segal E, Kostic AD, Patel CJ. The predictive power of the microbiome exceeds that of genome-wide association studies in the discrimination of complex human disease. bioRxiv. 2020; 2019.12.31.891978.

[93] Salosensaari A, Laitinen V, Havulinna AS, Meric G, Cheng S, Perola M, et al. Taxonomic signatures of long-term mortality risk in human gut microbiota. medRxiv. 2020; 2019.12.30.19015842. 10.1038/s41467-021-22962-y PMC8113604 33976176

[94] Sharon G, Cruz NJ, Kang DW, Gandal MJ, Wang B, Kim YM (2019). Human gut microbiota from autism spectrum disorder promote behavioral symptoms in mice. Cell.

[95] Wu H-J, Ivanov II, Darce J, Hattori K, Shima T, Umesaki Y (2010). Gut-residing segmented filamentous bacteria drive autoimmune arthritis via T helper 17 cells. Immunity..

[96] Wu M, Mcnulty NP, Rodionov DA, Khoroshkin MS, Griffin NW, Cheng J (2015). Genetic determinants of in vivo fitness and diet responsiveness in multiple human gut Bacteroides HHS public access. Science..

[97] Ridaura VK, Faith JJ, Rey FE, Cheng J, Duncan AE, Kau AL, et al. Cultured gut microbiota from twins discordant for obesity modulate adiposity and metabolic phenotypes in mice. Science. 2013;341. 10.1126/science.1241214. 10.1126/science.1241214 PMC3829625 24009397

[98] Lysenko AM, Stackebrandt E, Romanenko LA, Rohde M, Schumann P, Mikhailov VV (2002). *Psychrobacter submarinus* sp. nov. and *Psychrobacter marincola* sp. nov., psychrophilic halophiles from marine environments. Int J Syst Evol Microbiol.

[99] Maruyama A, Honda D, Yamamoto H, Kitamura K, Higashihara T (2000). Phylogenetic analysis of psychrophilic bacteria isolated from the Japan Trench, including a description of the deep-sea species *Psychrobacter pacificensis* sp. nov. Int J Syst Evol Microbiol.

[100] Bozal N, Montes MJ, Tudela E, Guinea J (2003). Characterization of several *Psychrobacter* strains isolated from Antarctic environments and description of *Psychrobacter luti* sp nov and *Psychrobacter fozii* sp nov. Int J Syst Evol Microbiol.

[101] Winn WC, Koneman EW (2006). Koneman’s color atlas and textbook of diagnostic microbiology.

[102] Bagley ST (1985). Habitat association of *Klebsiella species*. Infect Control.

[103] Ristuccia PA, Cunha BA (1984). Klebsiella. Infect Control.

[104] Podder MP, Rogers L, Daley PK, Keefe GP, Whitney HG, Tahlan K (2014). *Klebsiella species* associated with bovine mastitis in Newfoundland. PLoS One.

