# Supplementary figures and images for: Skin microbiome of beluga whales: spatial, temporal, and health-related dynamics
Source: Anim Microbiome. 2020 Oct 22;2:39. doi: 10.1186/s42523-020-00057-1 (PMC7807513; doi:10.1186/s42523-020-00057-1)

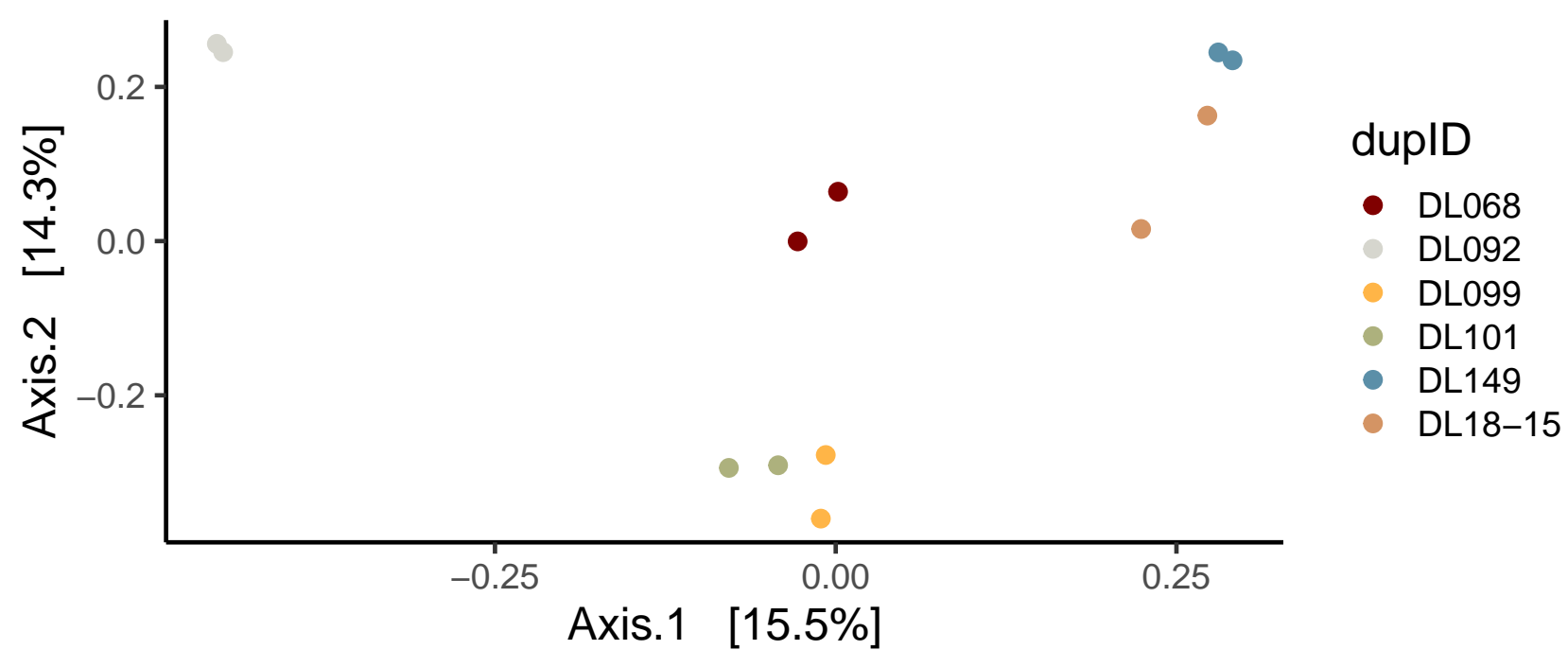

Supplement: Supplementary file 1 — Additional file 1: Supplemental Figure S1.. PCoA based on Bray-Curtis dissimilarity comparison of the epidermal microbiota of duplicate samples included in this study. [file 42523_2020_57_MOESM1_ESM.pdf]
